# Influence of the Ge–Chalcogenide Active Layer on Electrical Conduction in Self-Directed Channel Memristors

**DOI:** 10.3390/mi17040403

**Published:** 2026-03-26

**Authors:** Ahmed A. Taher, Kristy A. Campbell

**Affiliations:** Electrical and Computer Engineering, Boise State University, Boise, ID 83725, USA

**Keywords:** self-directed channel, SDC, memristor, Ge–chalcogenide (Ge-Ch), metal–chalcogenide (M-Ch), space charge limited conduction

## Abstract

The self-directed channel (SDC) class of memristors employs a multilayer architecture that is designed to enable robust Ag ion conduction, long cycling lifetime, and thermal stability. While several layers contribute to mechanical and chemical reliability, two layers primarily govern the electrical behavior: the amorphous Ge–chalcogenide active layer that is adjacent to the bottom electrode and the overlying metal–chalcogenide source layer. In this work, we investigate how the variation in the chalcogen species in these two layers influences switching characteristics in the pre-write regime, both in the pristine state and after a write/erase cycle, as well as the conduction behavior at room temperature. The devices were fabricated using Ge-rich chalcogenides containing O, S, Se, or Te, combined with SnS, SnSe, or Ag_2_Se metal–chalcogenide layers. The DC current-voltage measurements were analyzed using the standard linearization approaches to examine whether the transport behavior in the pre-write regime exhibits characteristics that are associated with Ohmic, Schottky, Poole–Frenkel, or space charge limited conduction. These measurements specifically probe the pre-write region of the *I-V* curve, where early ionic redistribution and structural rearrangement precede the abrupt formation of the conductive channels responsible for the resistive switching. The results show that the chalcogen composition strongly affects the threshold voltage, the resistance window, and the onset of field-enhanced transport, reflecting the differences in ionic distribution and channel formation dynamics. The results indicate that transport evolves with a bias and a compliance current, transitioning between regimes that are influenced by the interface injection and bulk-limited conduction, depending on the material stack. These findings clarify the role of chalcogen chemistry in governing the SDC switching behavior and provide guidance for the material selection in application-specific device design.

## 1. Introduction

Self-directed channel (SDC) memristors exhibit robust electrical characteristics, including a wide operational temperature range (250 K < T < 423 K), flexible switching speeds, including the ability to switch in sub-nanoseconds, depending on the device materials, and continuously variable resistance programming [1,2,3,4,5,6,7,8].

SDC-class memristors have attracted significant research attention due to their operational characteristics and simple design. The extensive studies on the SDC memristor have progressively clarified its electrical behavior, switching mechanisms, and application potential [9,10,11,12,13,14,15,16,17,18,19,20]. The early studies established the temperature dependence of its resistance states [2,7] and characterized low resistance conductivity over a range of switching frequencies and temperatures [8,10], providing an insight into the thermal activation of the transport processes. The broader analyses of memristor characteristics, material complexity, and functionality further positioned the SDC within the wider landscape of resistive switching devices [11]. Building on this foundation, the SDC has been utilized in the development of write algorithms that address and minimize device variability [13] and in communication circuits, such as binary phase-shift keying (BPSK) systems [14], highlighting its versatility across application domains. Additional investigations have demonstrated the efficient read operations that exploit the transient voltage responses in current-driven ReRAM cells, including SDC-type devices [5], that examined the activation energies associated with conductive channel formation and degradation [16] and proposed new memory architectures based on SDC technology [9]. The complementary modeling of sinusoidally driven devices [17] and studies of variability in commercial SDC implementations [18] have further strengthened the empirical and theoretical understanding that supports the continued optimization of SDC-based memory technologies. Moreover, SDC devices have demonstrated potential in diverse applications, including non-volatile memory, neuromorphic computing, artificial synapses for spike timing-dependent plasticity [19] and true random number generator circuits [20].

**Figure 1:**
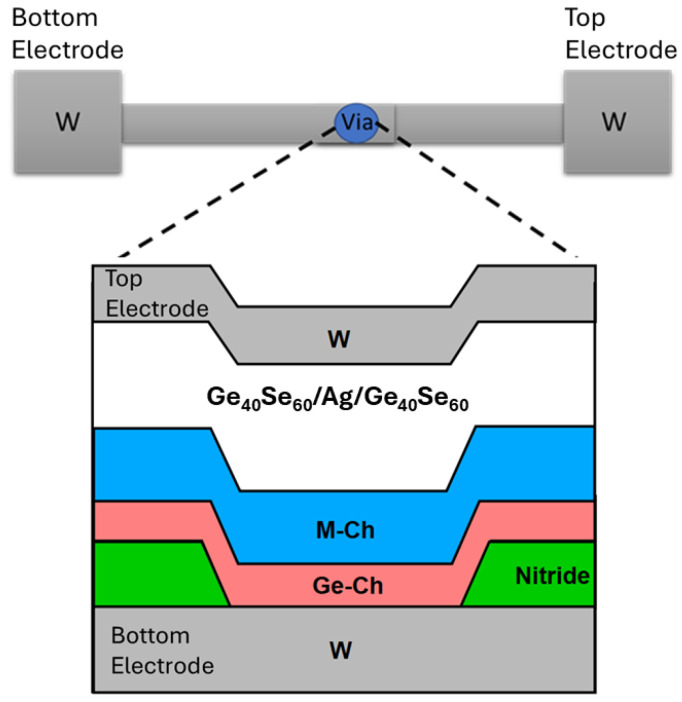
The schematic illustration of the SDC device structure showing a top-down view (**top**) and a cross-sectional view (**bottom**) of the active region. The active device layers are located within the via that is indicated in the top-down view.

The basic structure of an SDC class device is shown in Figure 1. The use of a germanium–chalcogenide (Ge-Ch) layer, Ge_40_Se_60_, and a metal–chalcogenide (M-Ch) layer, SnSe, in the SDC device to achieve variable resistance states, along with the benefits of each layer, was previously described [1]. The influence of the type of metal in the M-Ch layer, M-Se, on the SDC device operation was specifically examined by replacing the SnSe layer with other M-Ch materials [3].

Because the SDC device operation arises from the coupled interaction between the amorphous Ge-Ch active layer and the M-Ch layer during the channel formation and subsequent channel use, the effect of the material selection cannot be isolated to a single layer parameter. Consequently, this work examines a range of material permutations in order to identify the empirical trends in the switching behavior and transport evolution across the multilayer device stack. The goal is to determine how the combinations of Ge-Ch and M-Ch materials influence the transport evolution and channel formation in SDC devices. Accordingly, this work investigates how variations in both the Ge-Ch active layer and the M-Ch source layer influence the switching characteristics and pre-write transport behavior in the SDC memristors.

To examine these effects, devices are fabricated with the conventional Ge_40_Se_60_ layer and are replaced by compositionally similar, Ge-rich chalcogenides that incorporate S, Te, or O. In parallel, the M-Ch layer is varied among SnS, SnSe, and Ag_2_Se. The resulting devices are then characterized electrically under direct-current (DC, quasi-static) conditions [21,22,23,24,25,26,27].

## 2. Materials and Methods

The samples used to investigate the effect of the active layer composition on the device functionality are listed in Table 1. This study included a variation of the chalcogenide atoms (O, S, Se, and Te) in the Ge-Ch active layer, as well as a variation of the chalcogenide and metal type in the M-Ch layer, and, in some cases, the thickness of the M-Ch layer.

The SDC devices were fabricated in the Idaho Microfabrication Laboratory at Boise State University on 100 mm p-type wafers in a stacked layer structure (Figure 1) with a via that defines the device’s size at approximately 1 μm in diameter. The Ge-Ch layer was deposited either by thermal evaporation (Ge-S) using a CHA 600 thermal evaporator (CHA Industries, Fremont, CA, USA)or by sputtering (all other Ge-Ch materials) using an ATC Orion 5 UHV Magnetron sputtering system (AJA International, Hingham, MA, USA). All wafers received a brief Ar^+^ pre-sputter clean of the bottom electrode (W) just before the first layer (Ge-Ch) was deposited. Following the pre-sputter clean, the Ge-S samples were moved to the thermal evaporator for the Ge-S deposition, while all other Ge-Ch sample films were sputtered. Following the deposition of the Ge-Ch layers, the remaining SDC device layers were sputtered. The entire stack structure from the bottom to the top electrode was W/Ge-Ch/ M-Ch/Ge_40_Se_60_/Ag/Ge_40_Se_60_/W. All of the layers were sputter deposited in situ, except for the Ge-S, which had an air-break prior to and after the Ge-S evaporation. The final wafers were etched using a Veeco ME1001 ion-mill (Veeco Instruments Inc., Plainview, NY, USA).

The data analysis, plotting, and curve fitting were performed using IGOR Pro 9 (WaveMetrics, Inc., Lake Oswego, OR, USA).

To enable controlled comparisons, the samples are organized into groups in which only one structural parameter is varied while the remaining layers are held constant. These comparison groups allow for the individual influence of the Ge–chalcogenide composition, the metal–chalcogenide layer, or the M–Ch thickness to be evaluated separately, Table 2. This sample set is a continuation and complement to our prior work [1,2,3], which studied the baseline SDC structure operation [1], the effect of the type of metal ion source [2], and the metal type in the M-Ch layer [3].

### Quasi-Static DC Measurements at Room Temperature

**Figure 2:**
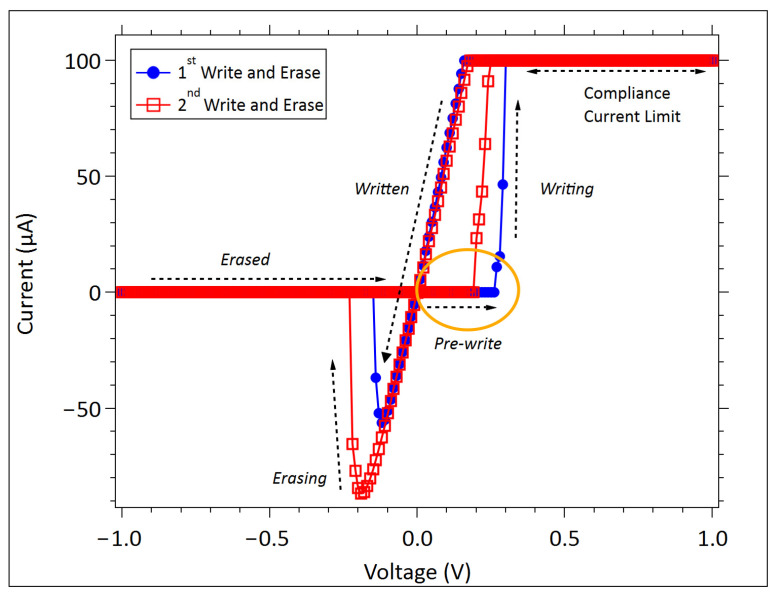
An example of the first and second sweep *I-V* curves from an SDC-class device. The *I-V* curves are divided into segments labeled: pre-write, writing, written, erasing, and erased. The analysis (pre-write) region is circled.

The quasi-static DC measurements were performed using a Keysight B1500A parameter analyzer (Keysight Technologies, Santa Rosa, CA, USA), which was operated in a voltage-sourcing mode while recording the resulting current. Each measurement consisted of two complete write–erase sequences: a first write and erase cycle (write 1, erase 1), followed immediately by a second write and erase cycle (write 2, erase 2), Figure 2.

We define five distinct voltage segments in an SDC-class device *I-V* curve, as labeled in Figure 2. These segments are as follows:

1. Pre-write: The region in which the device begins its transition from the initial high resistance to a lower resistance; this segment is the focus of the conduction mechanism analysis in this study.
2. Writing: The rapid transition region where the device switches toward a lower resistance state.
3. Written: The resistance state following a write operation, usually exhibiting Ohmic behavior.
4. Erasing: The region where the application of negative potential initiates a device’s return toward the higher resistance state.
5. Erased: The region after the completion of the erase sweep, in which the device typically reaches its maximum resistance, determined by the microscopic configuration of the conductive channels.

The write sweeps were executed as double sweeps from 0 → +1 → 0 V, and the erase sweeps as double sweeps from 0 → −1 → 0 V. The initial resistance (*R*ᵢ) was measured at +20 mV before any switching event occurred (post-fabrication, pristine device), ensuring that the device was measured in its undisturbed initial resistance state. After each write sweep, the written resistances (*R_w_*_1_ and *R_w_*_2_) were extracted at −10 mV during the following erase sweep, prior to the device erasing. The erased resistances (*R_e_*_1_ and *R_e_*_2_) were measured at −0.18 V during the erased segment.

A compliance current, *I_cc_*, was used during the measurement to prevent the device from being damaged during the transition to the low resistance state. The low and high compliance currents of 1 µA and 100 µA, respectively, were used to assess how the available current during the write sweep affects the programmed resistance and threshold voltage. Table 1 lists the number of devices from each sample measured at each compliance current.

The compliance-limited region is also denoted on the *I-V* curve in Figure 2; however, this portion reflects the instrument clamping current at the compliance limit rather than the device’s response and is therefore not considered an operational segment.

The parameters extracted from the DC sweep data also include the first and second write threshold voltages, *V_th_*_1_ and *V_th_*_2_, respectively. These are the voltages at which ‘significant’ conduction occurs.

The charge transport in the pre-write segment of a pristine device (defined as post-fabrication prior to any testing) is typically classified as either interface-limited or bulk-limited [21,22]. In SDC devices, resistive switching occurs through a field-driven incorporation of metal ions from the M-Ch layer into the Ge-Ch active layer, resulting in the formation of conductive channels within the amorphous matrix. During the pre-write segment, the applied electric field initiates ion redistribution and the associated redox reactions, which gradually modify the local bonding structure and electronic landscape of the Ge-Ch layer. As the voltage increases, these structural changes promote the nucleation and growth of conductive pathways. Once a sufficient network of conductive channels is established, the device undergoes the abrupt writing transition to a lower resistance state. Therefore, the conduction behavior that is analyzed in this work reflects the transport processes occurring during this evolving channel formation stage [1,2,3]. The common interface-limited mechanisms, which describe the current transport across the electrode–chalcogenide interface, that were studied with these devices include the Schottky thermionic emission model and Fowler–Nordheim (F-N) tunneling. The bulk-limited mechanisms, which describe the charge transport within the chalcogenide layer bulk, include the Poole–Frenkel (P-F) emission and a space charge limited conduction (SCLC).

In a basic single-layer chalcogenide two-terminal device, without ion transport, the Schottky thermionic emission model often dominates the interface-limited conduction at low voltage biases. The carrier transport is restricted by the interfacial potential barrier between the electrode and the chalcogenide layer. As the applied voltage increases, this barrier is progressively lowered, allowing the thermally activated carriers to surmount or tunnel through it. Once the barrier is sufficiently reduced, the conduction transitions to the bulk-limited mechanisms, such as P-F emission or SCLC. However, in the case of the SDC devices that are studied here, the M-Ch layer is a source of ions (from Ag and/or Sn) that can transport into the chalcogenide layer, even at low voltages. These ions continue to interact with the Ge-Ch layer and further modify the evolving channel structure during the pre-write process. Thus, while the Schottky mechanism may be present prior to significant ion movement and structural rearrangement, it will be modulated by the effects of simultaneous structural changes and ion movement into the Ge-Ch layer. Upon writing, the SDC conductive channels are formed, resulting in the emergence of different conduction modalities.

To explore the possible conduction mechanisms in the pre-write segment of the SDC device *I-V* curve, the experimental *I-V* data were fitted using the linearized forms of the F-N, Schottky, SCLC, and P-F models, which is an approach that is commonly used for dielectrics and phase-change chalcogenide materials [21,22]. A brief description of each investigated model and its governing equations is provided below.

Fowler–Nordheim tunneling: The electron wave function may penetrate the thin potential barrier when the applied electric field is sufficiently large. The current density for F-N tunneling is often expressed as follows:

(1) $$J=q^{2}E^{2}/(8\pi h\phi_{B})exp(-8\pi {\left(2qm^{*}\right)}^{1/2}{\phi_{B}}^{3/2}/(3hE))$$

where *J* is the current density (J=I/A), *E* is the electric field, *q* is the electron charge, ℎ is Planck’s constant, $m^{*}$ is the effective mass of the electron, and $\phi_{B}$ is the tunnel barrier height.

To evaluate whether F-N tunneling is present, ln(*I*/*V*^2^) is plotted against 1/*V*. A linear relationship indicates the possibility that F-N tunneling conduction is present, whereas the absence of linearity suggests that this mechanism is minimal or not contributing. It is noted that the presence of linearity does not ensure the presence of F-N tunneling, but that additional experiments (such as those conducted for temperature dependence) are necessary to conclude that it is present.

Schottky thermionic emission: The Schottky thermionic emission is a thermally activated process in which electrons from the electrode overcome the field-lowered interfacial barrier and are emitted into available energy states of the chalcogenide. The relationship for the Schottky emission is given by Equation (2):

(2) $$J=A^{*}T^{2}exp\left[-q(\phi_{B}-\sqrt{qE/4\pi \varepsilon_{i}})/kT\right]$$

Constants are as follows: $A^{*}$ is the effective Richardson constant, $\varepsilon_{i}$ is the insulator permittivity, $\phi_{B}$ is the Schottky barrier height, and *k* is Boltzmann’s constant. *q* and *T* are defined as they are in Equation (1).

To assess whether the Schottky thermionic emission contributes to conduction, the data are analyzed using an ln(*I*) versus $\sqrt{V}$ plot. The presence of a linear region in this plot is indicative of Schottky thermionic emission behavior.

Space charge limited conduction: This occurs when the current is dominated by carrier injection from an Ohmic contact and the resulting space charge within the dielectric further limits the current flow. In a log–log plot of the *I-V* characteristics, three regions are typically observed: an Ohmic region at low voltages, a trap-filled-limit (TFL) region where traps become saturated, and a trap-free (Child’s law) region at higher voltages, as described by Equations (3a)–(3c).

(3a) $$J_{ohm}=qn_{o}\mu V/d\;\;\;\;\;\;(Ohm’s\;law\;region)$$

(3b) $$J_{TFL}=9/8\varepsilon \theta \mu V^{2}/d^{3}\;(Trap-filled\;limit\;region)$$

(3c) $$J_{Child}=9/8\varepsilon \mu V^{2}/d^{3}\;\;\;\;(Child’s\;law\;region)$$

where q is the elementary charge, $n_{0}$ is the equilibrium carrier density, μ is the carrier mobility, d is the dielectric thickness, ε is the permittivity, and θ is the ratio of free total carriers (accounting for the trap occupancy).

P-F emission: This occurs when a strong electric field reduces the Coulombic potential barrier that traps electrons within an insulating or semiconducting material. The lowering of this field-assisted barrier enables the thermal excitation of trapped electrons into the conduction band, thereby increasing the current. The current density can be expressed as

(4) $$J=q\mu N_{t}E\exp\left[-q\left(\phi_{t}-\sqrt{qE/\left(\pi \varepsilon_{r}\varepsilon_{o}\right)}\right)/kT\right],$$

where *µ* is the carrier mobility, $N_{t}$ is the density of the trapped charge centers, $\phi_{t}$ is the trap barrier height, *E* is the applied electric field, $\varepsilon_{r}$ is the relative dielectric constant, and $\varepsilon_{0}$ is the permittivity of the free space (8.845 × 10^−14^ F/cm).

While the P-F and Schottky emission equations are similar, they differ in their physical origin. The P-F emission describes the bulk-limited conduction via field-enhanced emission from traps within the dielectric, whereas the Schottky emission represents the interface-limited conduction caused by the field lowering of the metal-insulator barrier.

In the present SDC devices, the amorphous structure and the presence of mobile ions such as Ag and Sn introduce significant spatial variations in the local electric fields, the dielectric properties, and the microscopic structure. These inhomogeneities render the quantitative extraction of physical parameters from linearized fits as unreliable. Consequently, the linearization plots, such as lnI vs. $\sqrt{V}$ and ln(I/V) vs. $\sqrt{V}$, are only employed here qualitatively to identify the similarities in the conduction behavior, without conclusively attributing the transport to a specific dominant mechanism. Because the internal structure of the SDC device evolves continuously during the pre-write process due to ion redistribution and structural rearrangement, the transport behavior does not correspond to a single steady-state electronic conduction model. Therefore, the classical transport models used here should be interpreted as qualitative indicators of the transport trends rather than a definitive identification of a dominant mechanism.

## 3. Results

### 3.1. DC I-V Measurement Parameter Analysis

**Figure 3:**
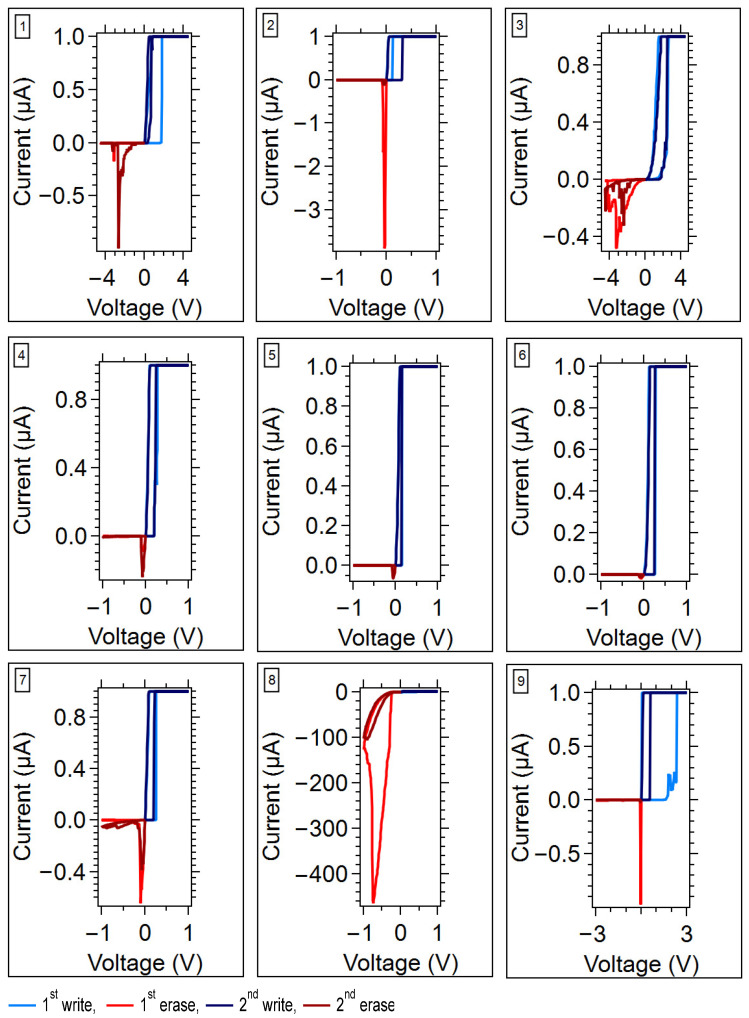
An example DC *I-V* for each sample with *I_cc_* = 1 µA. Each figure is labeled with the sample number. The sweeping directions follow the notation given in Figure 2. All of the measured device *I-V* curve plots are in the Supplementary Materials.

The DC *I-V* measurement plots for all the samples and device measurements are provided in the Supplementary Materials. One representative *I-V* curve from each sample for the case of 1 µA compliance current is shown in Figure 3. The extracted parameters from the *I-V* sweeps include the initial resistance (*R_i_*) (this is the pristine resistance), the first and second threshold voltages (*V_th_*_1_, *V_th_*_2_), the erase resistances (*R_e_*_1_, *R_e_*_2_), and the write resistances (*R_w_*_1_, *R_w_*_2_). All of the *I-V* curves were used for parameter extraction. The extracted parameters were averaged, and the standard deviations were calculated.

**Figure 4:**
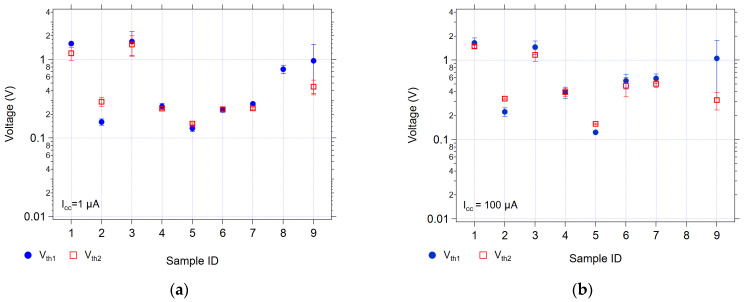
The average first and second threshold voltages for all samples using (**a**) 1 µA and (**b**) 100 µA as a compliance current. The error bars are one standard deviation.

**Figure 5:**
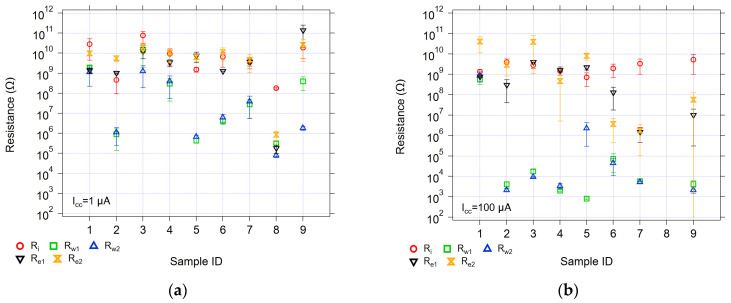
The average resistance *R_i_*, *R_e_*_1_, *R_e_*_2_, *R_w_*_1_, and *R_w_*_2_ for all samples tested with (**a**) 1 µA and (**b**) 100 µA compliance currents. The error bars are one standard deviation.

Figure 4 summarizes the average threshold voltages, and Figure 5 summarizes the average switching resistances, which were obtained under 1 µA and 100 µA compliance conditions. In general, the average threshold voltages for all the devices are below 2 V, with the GeS/SnS-based devices (Sample 1 and 3) having the highest threshold voltages and the GeSe-based devices having the lowest threshold voltages.

The initial resistance of all the device types exceeded 100 MΩ (Figure 5). This behavior was also observed for the first and second erase resistances following a write operation with a 1 µA compliance current for all the device types except the GeTe-based device (Sample 8). Sample 8 exhibited phase-change device behavior and therefore did not increase the resistance under a negative voltage sweep, unlike an SDC memristor [28,29].

The erase resistances in the case of a 100 µA compliance current did not follow the same trend as the 1 µA compliance current cases. Under these conditions, the GeSe-based devices more often exhibited erased resistances below 100 MΩ.

### 3.2. DC Conduction Mechanisms

The first positive voltage sweep probes charge the transport in the pristine a-Ge-Ch layer. In all the samples, this includes the conduction through the amorphous chalcogenide and some ion transport. Following the initial ion redistribution and formation of the self-directed channels, the conduction on the second positive voltage sweep includes both the electronic transport through the modified amorphous matrix and the field-driven ion motion within the newly established channels. An exception to this is Sample 8, which functions as a phase-change material after the first write incorporated Sn into the GeTe layer and created a phase-change ternary material [28,29]. Because the SDC device structure evolves during the pre-write process due to the ion redistribution and the structural rearrangement, the transport behavior may change as the voltage sweep progresses. Consequently, the following analyses are intended to identify similarities to classical conduction regimes rather than to assign a single dominant transport mechanism. Therefore, these analyses provide insight into how transport evolves during channel nucleation rather than establishing a uniquely constrained physical conduction model.

#### 3.2.1. F-N Tunneling

A linear fit to an $ln(I/V^{2})$ versus 1/V plot is used to investigate the possible F-N tunneling mechanisms. However, linearity does not necessarily indicate F-N tunneling and requires further studies, such as temperature dependence measurements, to conclusively assign the F-N tunneling mechanism. In our measurements, if we only consider the bulk Ge-Ch layer thicknesses, the corresponding electric fields across the devices would be on the order of a few hundred kV/cm, which is significantly below the threshold field (typically >5 MV/cm) observed for F-N tunneling in amorphous materials. In the SDC device, with Ag accumulation in the channels, it is reasonable to expect that electron tunneling could occur between conductive ‘islands’ within the channels in the Ge-Ch layer, as these are expected to be adjacent to each other [1]. However, no devices displayed any linear fit regions in this plot, with the exception of Sample 9, which had negligibly small linear regions, and these were at fields less than 400 kV/cm (see Supplementary Materials).

#### 3.2.2. Schottky Thermionic Emission

**Figure 6:**
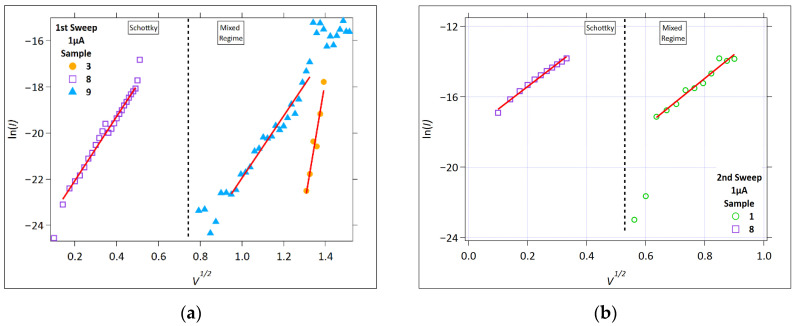
The Schottky plot (ln(*I*) versus $\sqrt{V}$) for *Icc* = 1 µA: (**a**) First positive sweep and (**b**) Second positive sweep. The solid red lines represent the linear fit.

**Figure 7:**
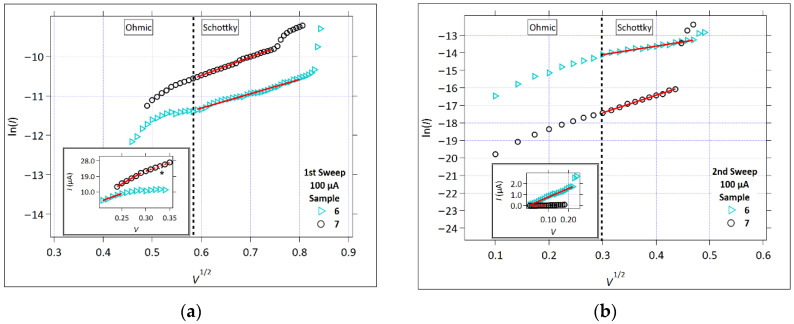
The Schottky plot (ln(*I*) versus $\sqrt{V}$) for *Icc* = 100 µA: (**a**) first positive sweep and (**b**) second positive sweep. The red lines represent the linear fit to the region of the data representing Schottky conduction. The inset graph in each plot shows an *I-V* plot of each sample with a linear fit in red, indicating an Ohmic response at the lower E fields. The * in the inset plot in (**a**) indicates a region where the slope changes from the lower voltage region. The vertical black dashed lines represent an arbitrary separation between possible conduction regions.

Figure 6 and Figure 7 show the ln(*I*) versus $\sqrt{V}$ Schottky plots for all the samples that exhibited linearity for the 1 µA and 100 µA cases, respectively.

**Figure 8:**
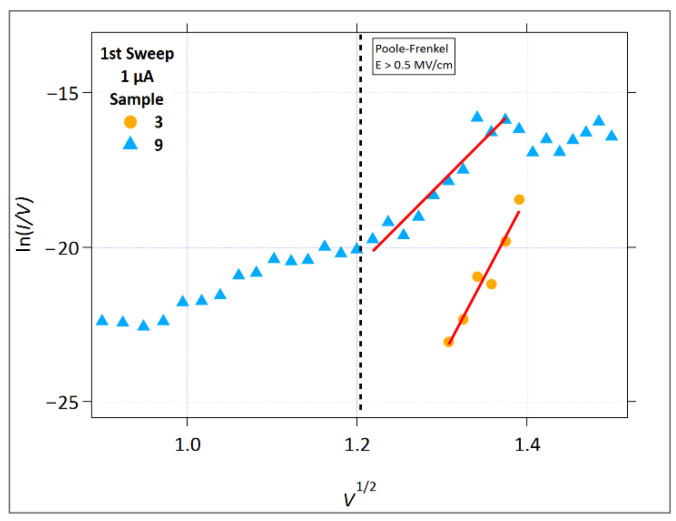
The P-F plot (ln(*I/V*) versus $\sqrt{V}$). The first positive sweep; *Icc* = 1 µA. The solid red lines represent the linear fit to the region of the data fitting the P-F emission.

During the first positive sweep, linearity is observed for Samples 3, 8, and 9 (Figure 6a) for the 1 µA compliance current measurements. It is observed for Samples 1 and 8 in the second sweep (Figure 6b). Despite the observed linear fits for these samples, Sample 8 shows behavior that is most consistent with the Schottky-type conduction. The other samples exhibit linearity at higher electric fields and appear in the P-F analysis (Figure 8), suggesting that the observed behavior reflects a field-enhanced transport that may involve both interface injection and trap-assisted bulk conduction rather than a uniquely identifiable mechanism.

Figure 7 shows the linear fit of the data obtained at a 100 µA compliance current, and only the data above 1 µA are included in this plot. Samples 6 and 7, which are Ge_40_Se_60_/SnSe-based samples that differ by the thickness of the SnSe layer (900 and 500 Å, respectively), both appear to exhibit Schottky conduction in the pre-write region. It is worth noting that the linearity extends over the range of 0.6–0.8 V^1/2^ for the first write and 0.3–0.45 V^1/2^ for the second write. This is above the threshold voltage range for the 1 µA compliance current case, shown in Figure 6. However, the threshold voltages are higher in these two samples for the 100 µA current compliance, as can also be seen in the *I-V* data for all the samples measured (Supplementary Materials).

#### 3.2.3. P-F Emission

Figure 8 shows the P-F plot (ln(*I*/*V*) versus $\sqrt{V}$) for the measurements that showed linearity at high E-fields, Samples 3 and 9. The P-F was observed only in these samples during the first positive sweep, at *I_cc_* = 1 µA. The two samples were also identified in the first sweep as *I_cc_* = 1 µA in the Schottky plot (Figure 6a) and were labeled as ‘Mixed Region’. These samples exhibit linearity above the electric fields of 0.5 MV/cm, whereas the Schottky conduction is commonly expected to be found below 0.1 MV/cm. There were no clear linear regions in the *I_cc_* = 100 µA plots.

#### 3.2.4. Space Charge Limited Conduction

**Figure 9:**
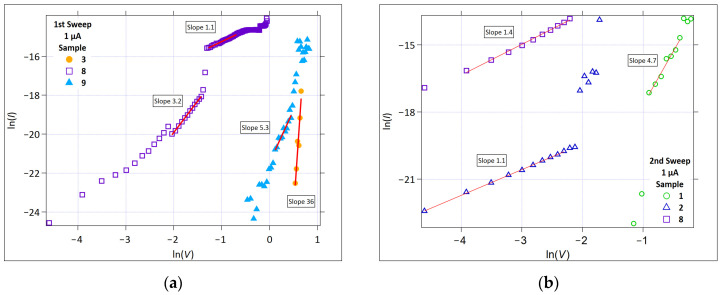
The SCLC plot (ln(*I*) versus ln(*V*)), *Icc* = 1 µA. (**a**) The first positive sweep. (**b**) The second positive sweep. The solid red lines represent the linear fit regions.

Figure 9 shows the ln(*I*) versus ln(*V*) plot that was used to investigate the presence of SCLC. For the 1 µA data, Samples 3, 8, and 9 exhibited linearity in the first write sweep. In the second write sweep, the samples exhibiting linearity were 1, 2, and 8. The slope of the line fit for Sample 3 is 36, which is not a meaningful indication of SCLC. A slope this high is more likely to be indicative of a switching transition region, likely a field-driven structural/ionic modification.

**Figure 10:**
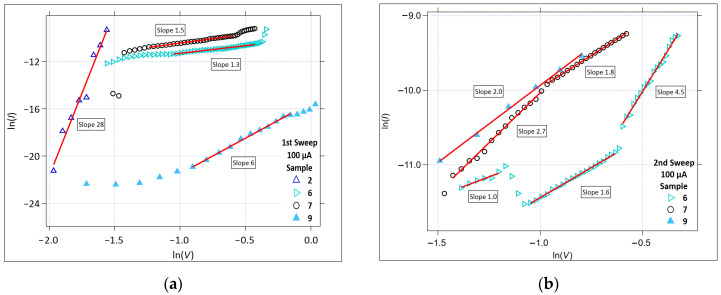
The SCLC plot (ln(*I*) versus ln(*V*)), *Icc* = 100 µA. (**a**) The first positive sweep. (**b**) The second positive sweep. The solid red lines represent the linear fit regions.

Figure 10 shows the SCLC plots for the 100 µA compliance current data. At the higher compliance current, both higher voltages and higher currents were reached, resulting in different samples with linearity in this plot. In the first sweep, at low sweep voltages, Sample 2 exhibits linearity with a slope of almost 30. As for Sample 3 at a 1 µA compliance current (Figure 9), the slope is too high to indicate the SCLC. Again, it likely indicates that there is a significant field-driven structural and/or ionic modification in the Ge-Ch layer.

Only samples 4 and 5, Ge_40_Se_60_/SnS and Ge_40_Se_60_/Ag_2_Se, respectively, did not show linearity in any of the conduction mechanism plots.

## 4. Discussion

The resistive switching process in the SDC devices is linked to the formation and evolution of conductive channels within the Ge-Ch active layer. This process has been described previously [1,3] and differs between the first write of a pristine device and the subsequent write operations. During the first pre-write segment, as the applied voltage increases, the metal ions originating from the M-Ch layer migrate into the amorphous Ge-Ch matrix and participate in redox reactions that locally modify the bonding structure and electronic transport pathways, leading to the nucleation of self-directed channels. This progressive ionic redistribution and structural rearrangement alter the local electric field distribution and trap the landscape, eventually producing the conductive pathways responsible for the abrupt writing transition. Consequently, the transport characteristics that were observed in the pre-write region of the first write sweep reflect the early stages of channel nucleation and growth, and the differences between the material systems provide qualitative insight into how composition may influence the dynamics of the channel formation. In subsequent write cycles, the channels are already established and serve as preferred pathways for ion and electron transport. Under similar voltage and current conditions, these channels are reused, while increases in the maximum applied voltage or current limit can lead to further channel expansion or to the formation of additional conductive paths.

### 4.1. Comparison of DC I-V Parameters and Conduction Mechanism Analysis by Group

The grouped comparison approach isolates the individual material parameters, allowing the influence of the active layer composition, source-layer composition, and thickness to be examined independently.

#### 4.1.1. Group A: Ge-S/SnS, Samples 1 and 3

S is the only chalcogenide present in Samples 1 and 3 (Ge_42_S_58_/SnS). As shown in Figure 4, these samples exhibited the highest average threshold voltages under both compliance currents. Even though these samples share the same type of material stack, they were processed in different lots (at separate times) and have differences in their operational parameters. Ideally, processing materials under the same conditions should produce similar results, but the S-based devices were particularly prone to lot-to-lot and device-to-device variability.

Both samples have a similar range of average *V_w_*_1_ and *V_w_*_2_ values. However, an inspection of the resistances (Figure 5) shows that while both samples have similar resistance distributions at a 1 µA compliance current, the 100 µA compliance current data for Sample 3 shows a five order of magnitude difference between the written and erased resistances, ranging from 1 GΩ to 10 kΩ. Conversely, Sample 1 shows similar high resistances at both compliance currents.

The differences between Samples 1 and 3 are also obvious in the DC conduction analysis. Sample 1 only displayed linearity in the Schottky and SCLC second voltage sweeps in the 1 µA compliance current plots. Sample 3 had linear regions in the first sweep and had a 1 µA compliance current plot for Schottky, P-F, and SCLC. However, the SCLC linear fit slope (36) is not meaningful for SCLC, indicating instead that the sample is likely undergoing a transition (structural and ionic movement).

These observations indicate that the Ge_42_S_58_/SnS sample types are highly sensitive to subtle processing differences. Although Samples 1 and 3 have the same nominal stack, their electrical behavior indicates differences in their starting structure. The larger resistance window in Sample 3 between the initial/erased and the written values, and the fact that it shows Schottky and P-F linearity in the first sweep at 1 µA, indicate that the electric field begins altering carrier transport in this sample at relatively low stress (also supported by the linearity in the SCLC plot, with a high slope). This likely reflects the ionic redistribution or modification of the internal barriers at a lower electric field. In contrast, Sample 1 requires additional electrical stress before similar transport changes appear. Together, this suggests that small structural differences strongly influence how and when the conductive channel develops in the sulfur-based devices, directly affecting their transport and switching behavior.

#### 4.1.2. Group B: Ge-S/SnS and Ge-Se/SnS, Samples 3 and 4

These two samples differ only in the Ge-Ch layer; Sample 3 has S, and Sample 4 has Se. Both samples use Sn-S as the M-Ch layer. The Ge-S sample exhibited threshold voltages an order of magnitude higher than the Ge-Se sample. The resistances of both samples are similar under both the compliance currents, with the Ge-S sample having slightly higher resistances in the initial and erased states, and are about an order of magnitude higher in the written states. Sample 4 did not display linearity in any of the conduction mechanism plots, whereas Sample 3 did, as mentioned in Section 4.1.1 within the discussion of Group A.

#### 4.1.3. Group C: Ge-S/Ag_2_Se and Ge-Se/Ag_2_Se, Samples 2 and 5

In Sample 2, Ag_2_Se replaced the SnS layer in the Ge-S device, and a much lower threshold voltage was generally observed (0.2–0.3 V as opposed to 1–2 V observed in Sample 3). However, Sample 2 devices exhibited erratic erasing responses, often requiring significantly higher currents to erase (see Supplementary Materials). Sample 5, with Ag_2_Se above a Ge-Se layer, had threshold voltages slightly lower than those of Sample 2, at about 0.2 V.

Sample 2 and Sample 5 showed similar distributions of resistances at the 1 µA compliance current. At a 100 µA compliance current, Sample 5 has a higher *R_w_*_2_ (MΩ) compared to Sample 2 (kΩ).

Sample 2 exhibited linearity at voltages below 150 mV in the SCLC plot 1µA second positive sweep, with a slope of approximately one, which is an indication of Ohmic conduction. Sample 2 also had a linear region between about 0.35 and 0.9 V in the first write sweep for a 100 µA compliance current with a slope of approximately 5. This slope corresponds to trap-limited SCLC. Trap-limited SCLC occurs when injected carriers in amorphous material are statistically captured by localized energy states, leaving a small fraction of mobile carriers. With an increasing voltage, the traps become more filled, leading to an increase in the mobile carrier population. This appears in the SCLC plots as a linear region with a slope typically between two and six. This effect in the pre-write segment corresponds to the structural rearrangement and the presence of mobile ions within the Ge-S layer.

Sample 5 (Ge-Se/Ag_2_Se), which exhibits the lowest average threshold voltages and a moderate erase and written resistance separation window, does not show clear linearity in any of the standard conduction mechanism plots. This is likely because its transport is dominated by early and distributed ionic motion rather than a single steady-state electronic process. The lower threshold suggests that Ag redistribution begins at a relatively low field, which continuously modifies the layer’s chemical structure during the sweep. As a result, the device does not enter a stable, well-defined conduction regime when in a sufficient voltage range to produce linear behavior in any of the conduction plots. Instead, the transport appears to evolve gradually with bias, preventing a clear identification of a dominant classical mechanism.

#### 4.1.4. Group D: Ge-Se/SnS, Ge-Se/SnSe, Samples 4 and 7

For Samples 4 and 7, the threshold voltages were similar for both write sweeps (240 mV) at the 1 µA compliance current. At 100 µA, Sample 7 had a write threshold voltage of approximately 500 mV, which is about 100 mV higher than that of Sample 4 (400 mV). This change is to be expected if the Ge-Se structure is being modified through electric field-induced ion movement into the layer; the higher compliance current allows higher electric fields across the device. This results in a higher threshold voltage if there is a higher channel resistance during the voltage sweep. This is the case for the Ge-Se/SnSe device. A higher channel resistance during the pre-write segment as the voltage is increasing could be due to several factors. For example, either (1) less Sn is entering the Ge-Se in the case of SnSe; (2) less Ag (from the top electrode) is entering the Ge-Se channels; or (3) there are fewer parallel channels spanning the Ge-Se layer in the case of Ge-Se/SnSe than in the corresponding SnS-based device.

The resistances for Sample 4 at the 1 µA compliance current were all above 100 MΩ, whereas Sample 7 had a wider separation (> one order of magnitude) between high and low resistance groups. At the 100 µA compliance current, the resistance separation between high and low states for the SnS-based devices (Sample 4) was almost six orders of magnitude (from 10^3^ to 10^9^). The Sample 7 SnSe-based devices showed erase resistances that were three orders of magnitude lower (10^6^) than the SnS-based devices, with written resistances in the 10^4^ range.

#### 4.1.5. Group E: Samples 6 and 7

Samples 6 and 7 were fabricated with the same material stack (Ge_40_Se_60_/SnSe) but with different M-Ch layer thicknesses (900 or 500 Å, respectively) to evaluate the effect of M-Ch thickness on switching parameters. The results indicate that varying the SnSe layer thickness had no significant influence on the threshold voltages. The resistances that were obtained for the thicker SnSe-layered sample, Sample 6, showed higher erase resistance values than those of the thinner SnSe case (Sample 7).

Both samples exhibited a similar linear response in the Schottky and the SCLC plots for the 100 µA compliance current in the first and second sweeps. It is interesting to consider the definition for the pre-write segment. We assume that pre-write is the region prior to the channel formation (for the first write sweep), and prior to the abrupt (or significant) current flow in the second sweep. This is a straightforward definition when considering the case of a low compliance current, like 1 µA. Typically, the low current allows channels to form without significant heating or the excess incorporation of metal ions (especially from the Ag source layer). When the current is allowed to increase to a higher compliance value, the localized fields can result in material heating and reorganization, excess metal ion incorporation in the channels, and multiple channel formations, with the result of some conduction and ‘pre-writing’ prior to reaching the compliance current value. The conduction mechanisms in this case extend beyond what was present in the low compliance current case, so that the overall conduction mechanisms at 100 µA will include channel conduction from the channels formed at the low currents. This is most evident in this study when looking at the data for Samples 6 and 7, and their linear fits in the Schottky (Figure 7) and SCLC (Figure 10) plots. The Schottky plots show similar linearity for both samples up to the point where the low compliance current threshold voltage is reached, which is around 0.46 V^1/2^ (= 0.21 V). In the SCLC plots, linearity is absent until the voltage enters a region that can be reached with the higher compliance current, which is around 0.33 to 0.9 V.

The slopes of the SCLC plot lines for Samples 6 and 7, 100 µA in the first sweep, are 1.3 and 1.5, respectively. These are between the Ohmic and the trap-free conduction regions. In the second sweep, multiple linear regions are present. Sample 6 shows an Ohmic region at low voltages after the region is defined as switching in the low compliance current condition (from 0.22 to 0.30 V), with a slope of one, which is followed by a region of mixed Ohmic and trap-free conduction (from 0.36 to 0.54 V) with a slope of 1.5, which is then followed by a trap-limited conduction region (from 0.57 to 0.75 V) with a slope of 4.5. Sample 7 shows two linear regions, with the first having a slope of 2.7 and the second having a slope of 1.8. Both of these indicate trap-limited conduction, with the differences in the slope likely due to the structural modifications as the field is increased.

From this, we observe that, for the Ge_40_Se_60_/SnSe samples that switched using a higher compliance current, the device current is no longer primarily limited by carrier injection at the electrode interface but instead by charge buildup within the bulk of the Ge_40_Se_60_ layer. This behavior emerges after the initial low-current threshold event that is associated with early channel formation. Once the conductive pathways are established, increasing the compliance current can drive additional structural modification and metal incorporation into the Ge_40_Se_60_; however, the overall current becomes governed by bulk transport constraints rather than by the injection barrier.

#### 4.1.6. Group F: Ge_40_Se_60_/SnSe, GeTe/SnSe, and Ge_40_Se_60_+Ox/SnSe, as Well as Samples 6, 8, and 9

This group varies the Ge-Ch layer, keeping the M-Ch layer as SnSe. These samples run down the Group VI elements of the periodic table, from Se to Te to GeSe-Ox.

Sample 9, which includes oxygen in the active layer (GeSe-Ox), was compared with Sample 6 to assess the impact of oxygen incorporation on device switching. The addition of oxygen significantly affected the threshold voltages, both in magnitude (as high as 1 V) and consistency, showing the largest standard deviation among all of the samples. The resistances showed similar large distributions and inconsistencies from device to device in the oxygen-containing sample.

Comparing Samples 6 and 8, using SnSe as the M-Ch layer but using different Ge-Ch active layers, demonstrates the importance of the Ge-Ch layer on the device operation and the switching stability. Sample 8 did not exhibit a distinct second threshold voltage, displaying an Ohmic response and indicating crystallization of the GeTe layer (phase-change behavior) after the first write sweep. Additionally, there was a lack of erasing to a higher resistance upon the application of a negative voltage. 

## 5. Conclusions

This study demonstrates that SDC device behavior is strongly governed by the interaction between the Ge-Ch active layer and the M-Ch source layer, and that even subtle compositional changes significantly alter the channel formation dynamics and transport evolution. The sulfur-based systems exhibited the highest threshold voltages and pronounced sensitivity to processing variations, indicating that small differences in the initial structural or defect landscape strongly influence when and how the conductive channels develop. In contrast, selenium-based systems generally switched at lower voltages and showed a more gradual, field-driven transport evolution.

The conduction mechanism analysis further revealed that the transport behavior is not uniform across the material systems. In some stacks, field-enhanced electronic transport signatures appear prior to full channel formation, suggesting early modification of internal barriers. In others, particularly Ge-Se/Ag_2_Se, transport evolves continuously with bias and does not enter a well-defined steady-state regime, indicating that ionic redistribution dominates classical bulk-limited conduction. At higher compliance currents, especially in Ge_40_Se_60_/SnSe devices, the transport transitions toward bulk-influenced behavior after the initial channel formation are consistent with the charge buildup within the active layer rather than with injection-limited conduction.

Together, these results show that the threshold voltage, the resistance window, and the conduction behavior are not independent parameters but reflect how readily each material system undergoes field-driven structural and ionic modification. The stability, variability, and transport regime of the device are therefore directly tied to the chemical and structural response of the stack under bias. Because the SDC structure evolves dynamically during bias through ionic redistribution and structural modification, the conduction models discussed in this work should be interpreted as qualitative descriptors of transport behavior rather than the definitive identification of a single dominant conduction mechanism.

As a next step, we are investigating the continuous-wave sinusoidal frequency response of these devices to examine their behavior under periodic bias. Frequency-dependent hysteresis measurements will allow us to determine how quickly the conductive pathways respond to changing electric fields, how much energy is dissipated per cycle, and how stable and controllable the conductance states remain under repeated operation. These dynamic characteristics are critical for application selection: systems that maintain stable hysteresis at higher frequencies are better suited for memory and computing applications, while those exhibiting gradual and symmetric conductance modulation may be advantageous for analog or neuromorphic uses. The frequency response will therefore provide a practical basis for comparing material systems beyond what can be inferred from DC switching alone.

## Figures and Tables

**Table 1:** The sample identification and material composition.

Sample	Number of Devices Tested for Each *I_cc_* Group (1 µA/100 µA)	Ge-Ch	M-Ch ^#^
1 *	3/3	Ge_42_S_58_	500 Å SnS
2 *	3/3	Ge_42_S_58_	470 Å Ag_2_Se
3 *	3/3	Ge_42_S_58_	500 Å SnS
4	3/3	Ge_40_Se_60_	500 Å SnS
5	3/3	Ge_40_Se_60_	470 Å Ag_2_Se
6	3/3	Ge_40_Se_60_	900 Å SnSe
7	3/3	Ge_40_Se_60_	500 Å SnSe
8 *	4/0	GeTe	900 Å SnSe
9	3/5	Ge_40_Se_60_-Ox	1000 Å SnSe

* evaporated Ge-Ch films. All the others were sputtered. ^#^ All the M-Ch layers were evaporated except for Ag_2_Se, which was sputtered.

**Table 2:** The sample comparison groups.

Group	Sample Numbers	Description
A	1, 3	**Ge-S/SnS**: Same target materials, but different lots
B	3, 4	**Ge-Ch**: Ge-Se vs. Ge-S both with SnS
C	2, 5	**Ge-Ch**: Ge-Se vs. Ge-S both with Ag_2_Se
D	4, 5, 7	**M-Ch**: Ge_40_Se_60_ with SnS, Ag_2_Se, or SnSe
E	6, 7	**M-Ch thickness**: Ge_40_Se_60_ with 900 or 500 Å SnSe
F	6, 8, 9	**Ge-Ch**: Ge-Se, Ge-Te, Ge-Se+O, with 900 to 1000 Å SnSe

## Data Availability

The original contributions presented in this study are included in the article/Supplementary Material. Further inquiries can be directed to the corresponding author.

## Supplementary Materials

The following supporting information can be downloaded at https://www.mdpi.com/article/10.3390/mi17040403/s1, Figures S1–S9: DC data for all measured Sample 1 (through 9) devices; Figure S10: F-N plots for Sample 9.

## Abbreviations

The following abbreviations are used in this manuscript:

SDC	Self-directed channel
Ge-Ch	Germanium–chalcogenide
M-Ch	Metal–chalcogenide
DC	Direct-current or quasi-static
I_cc_	Compliance current
V_th1_	First write threshold voltage
V_th2_	Second write threshold voltage
R_i_	Initial resistance
R_e1_	First erased resistance
R_e2_	Second erased resistance
R_w1_	First written resistance
R_w2_	Second written resistance
SCLC	Space charge limited conduction
P-F	Poole–Frenkel conduction
F-N	Fowler–Nordheim tunneling
ReRAM	Resistance random access memory
