# Supplementary material for: Influence of the Ge–Chalcogenide Active Layer on Electrical Conduction in Self-Directed Channel Memristors
Source: Micromachines (Basel). 2026 Mar 26;17(4):403. doi: 10.3390/mi17040403 (PMC13118175; doi:10.3390/mi17040403)
Supplement: Supplementary file 1 [file micromachines-17-00403-s001.zip › micromachines-4205719-supplementary/micromachines-4205719-supplementary.pdf]

Supplementary Materials

Influence of the Ge–Chalcogenide Active Layer on Electrical Conduction in Self-Directed Channel Memristors

Ahmed A. Taher and Kristy A. Campbell *

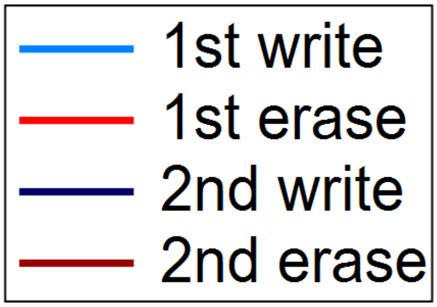

Legend for the data figures.

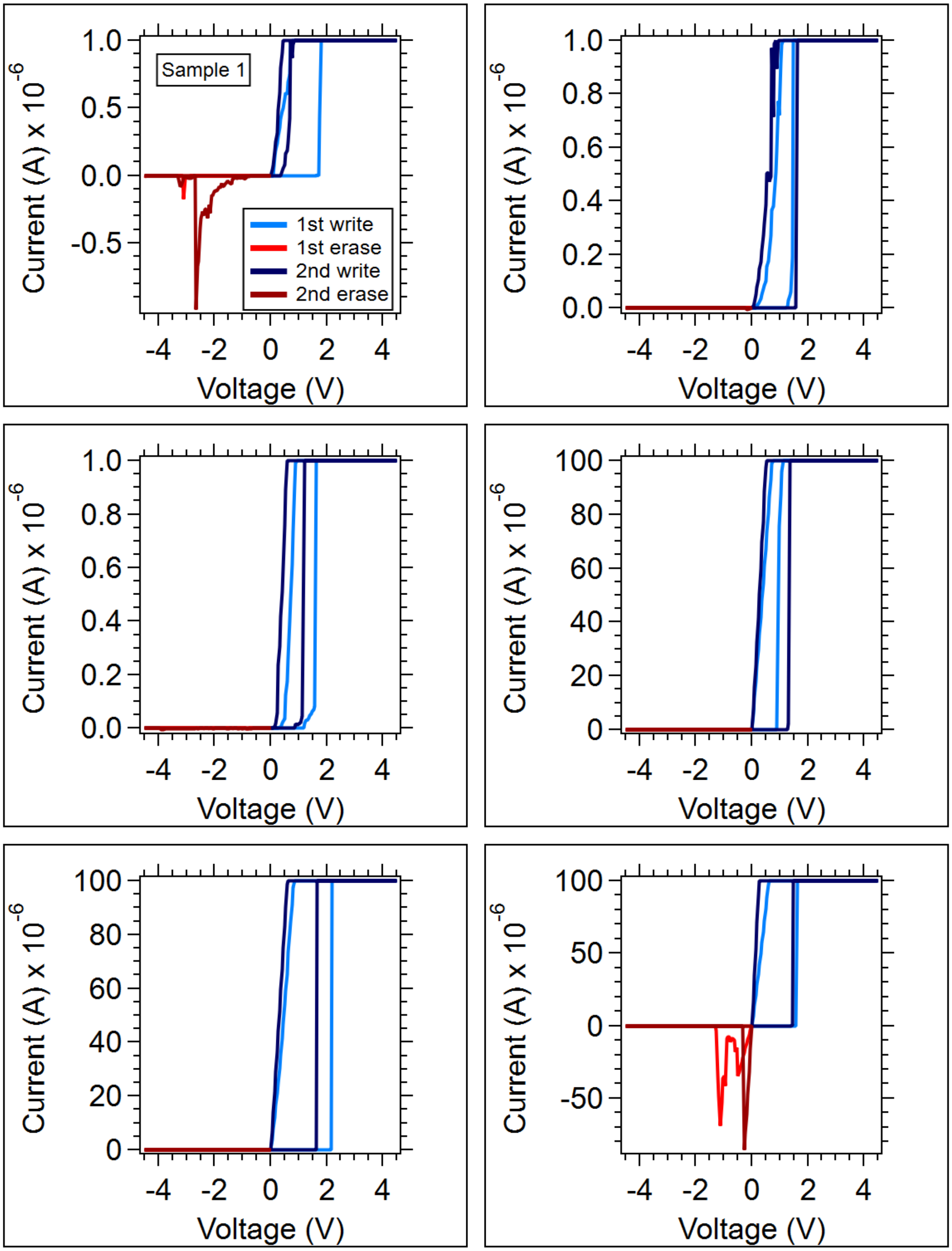

Figure S1. DC data for all measured Sample 1 devices

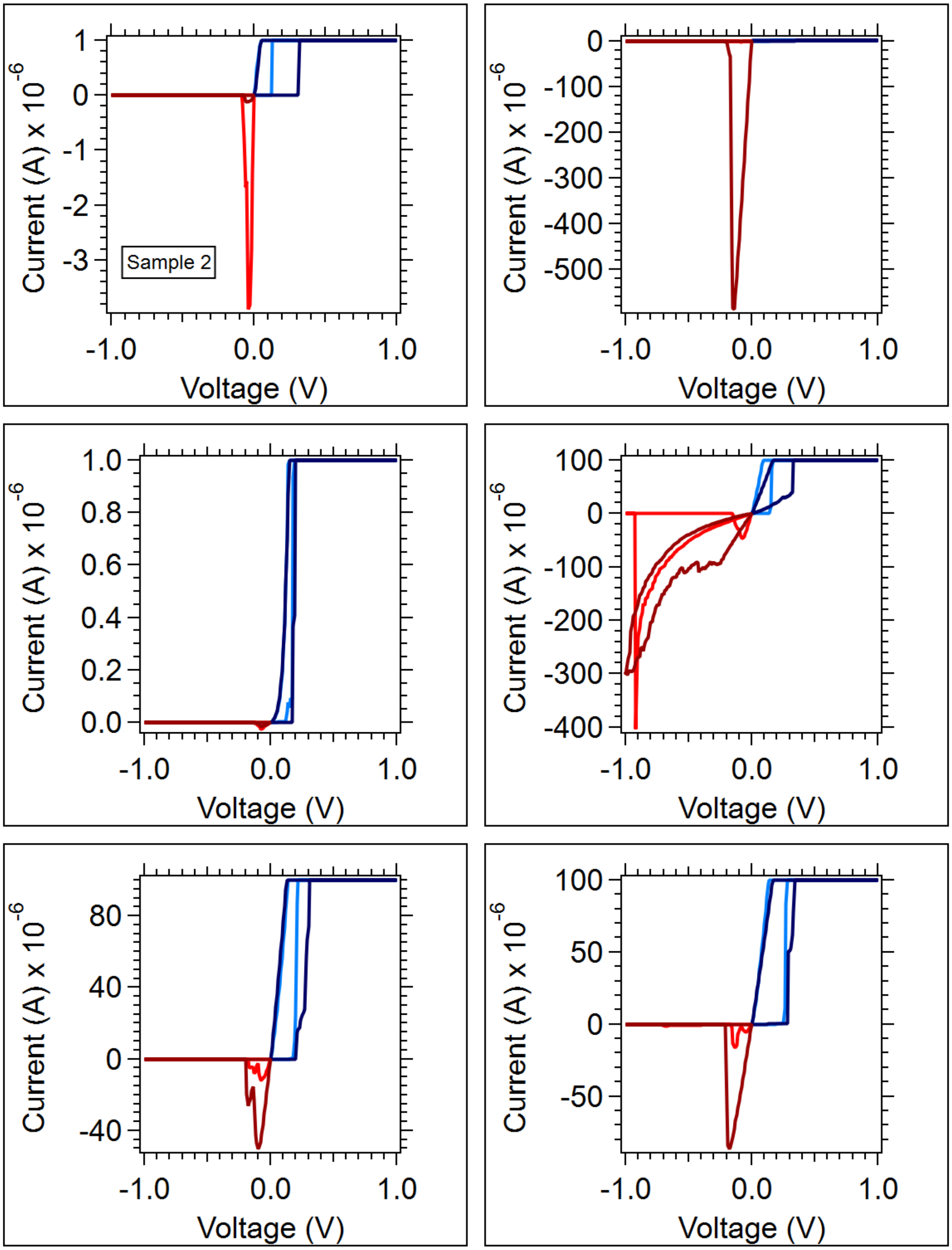

Figure S2. DC data for all measured Sample 2 devices

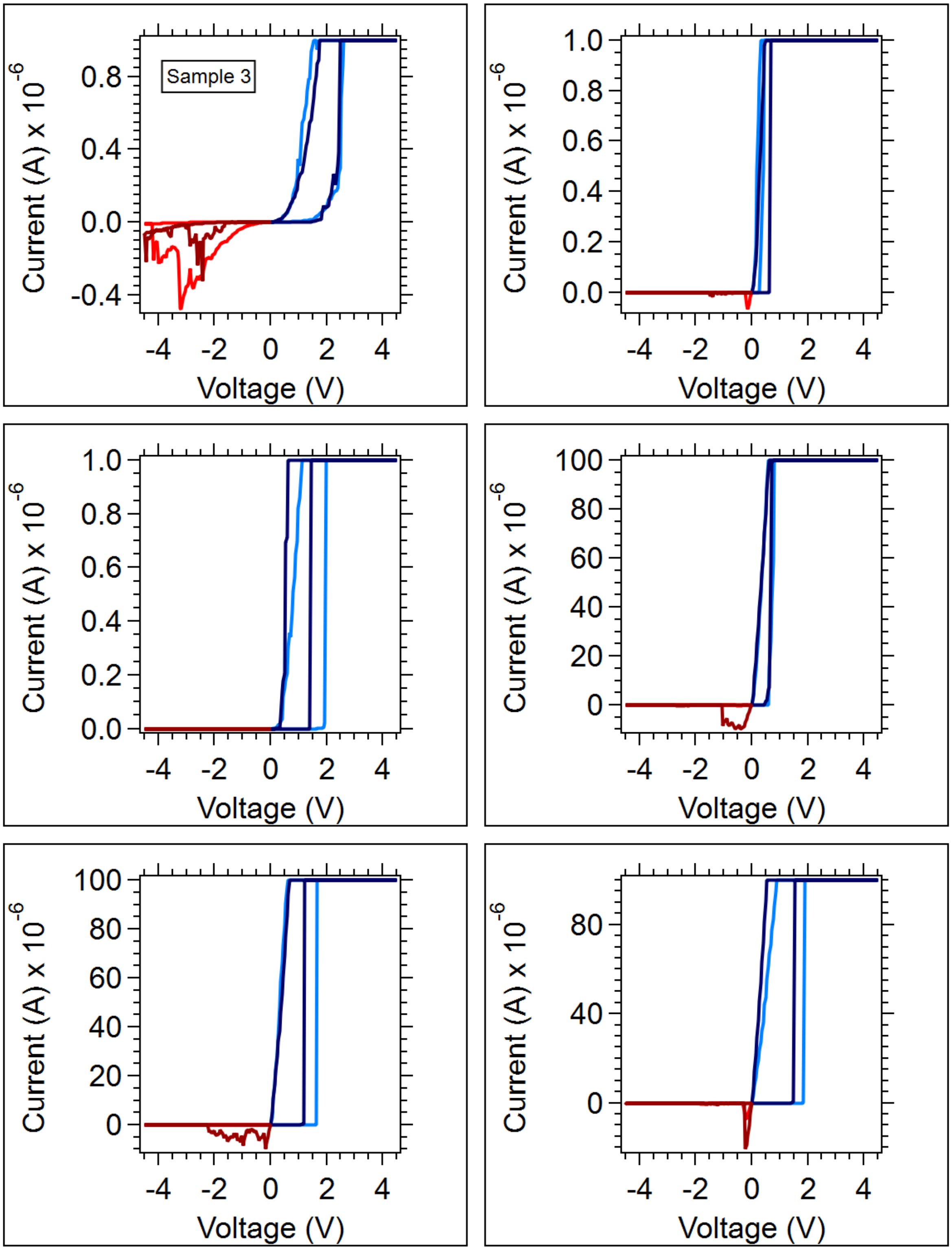

Figure S3. DC data for all measured Sample 3 devices

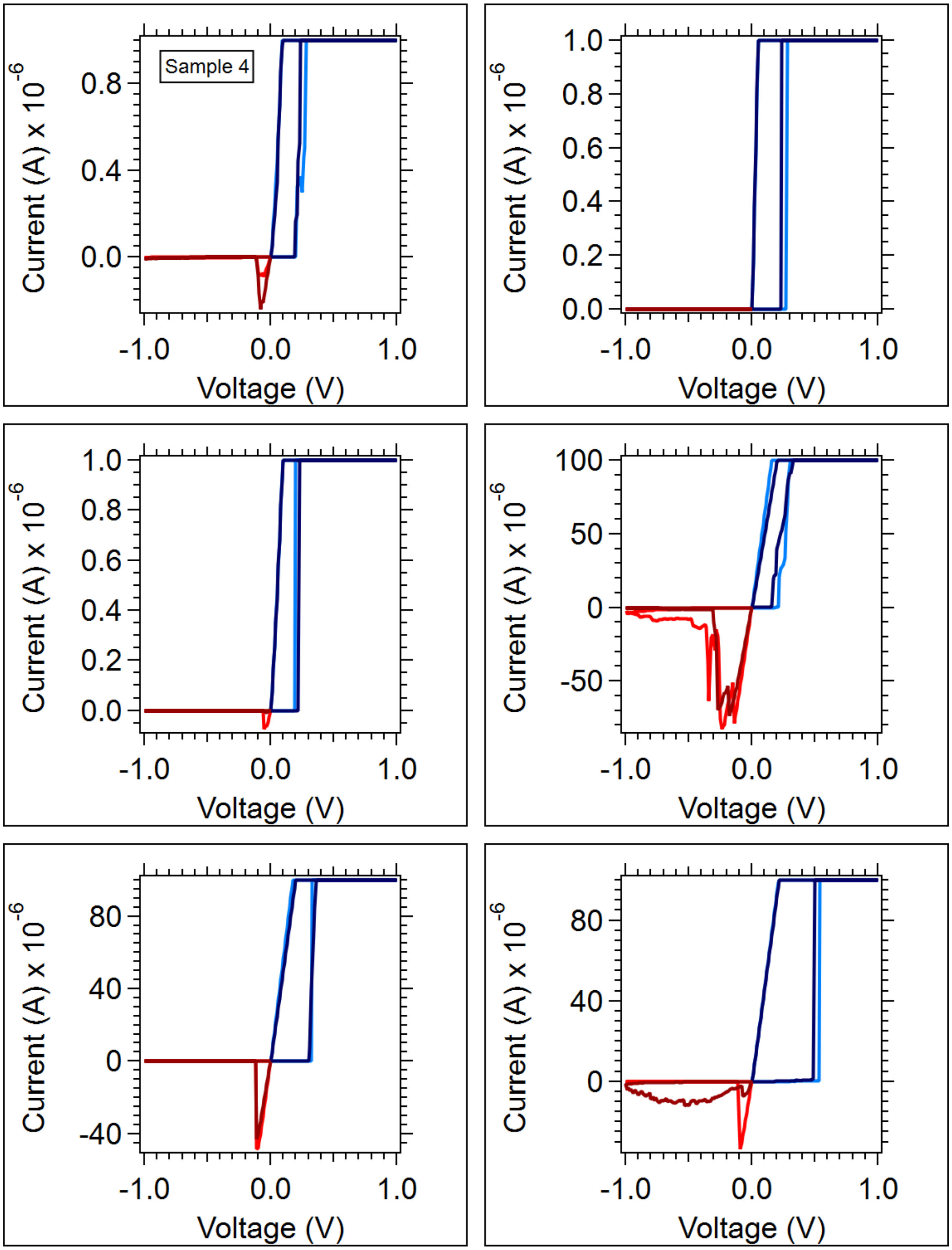

Figure S4. DC data for all measured Sample 4 devices

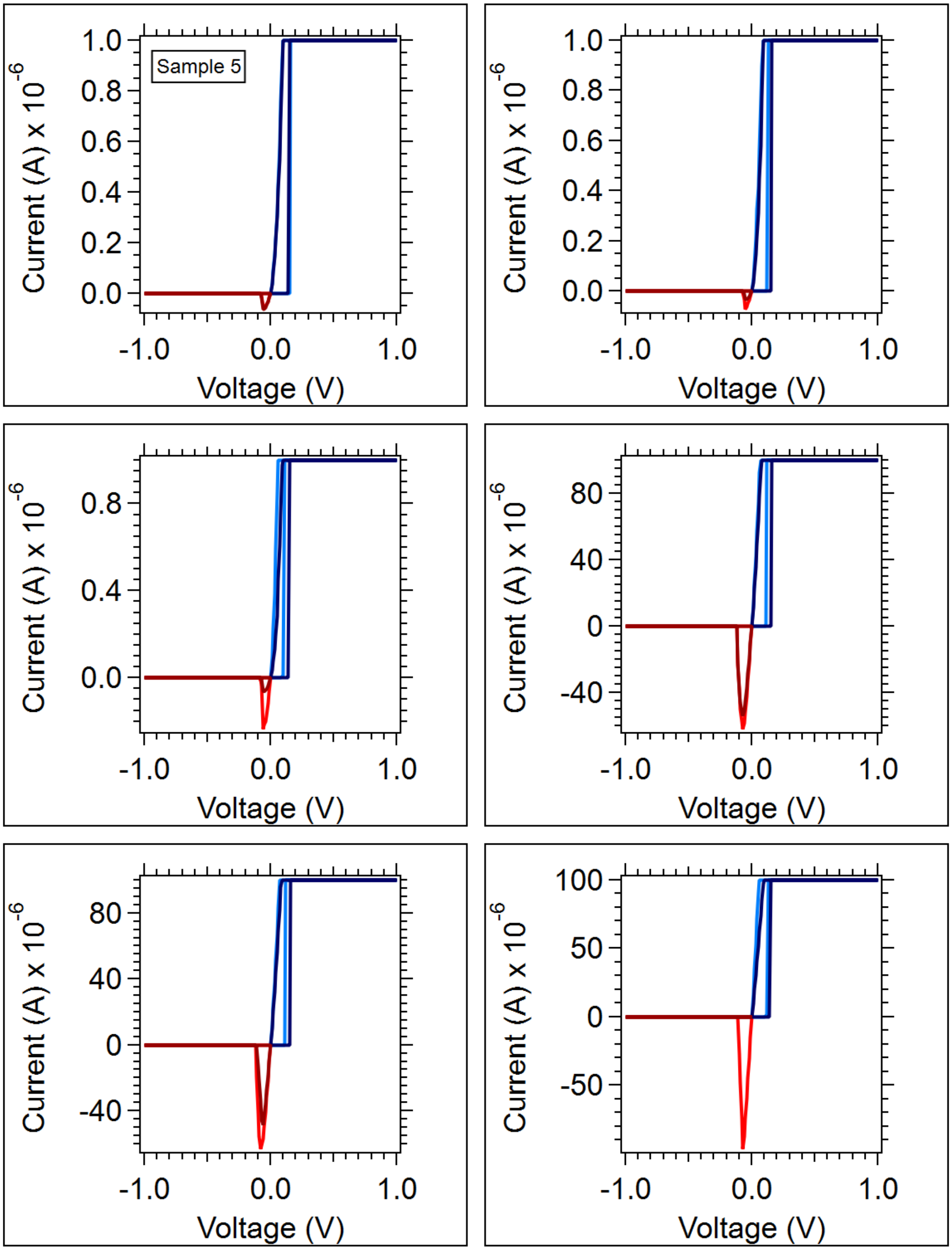

Figure S5. DC data for all measured Sample 5 devices

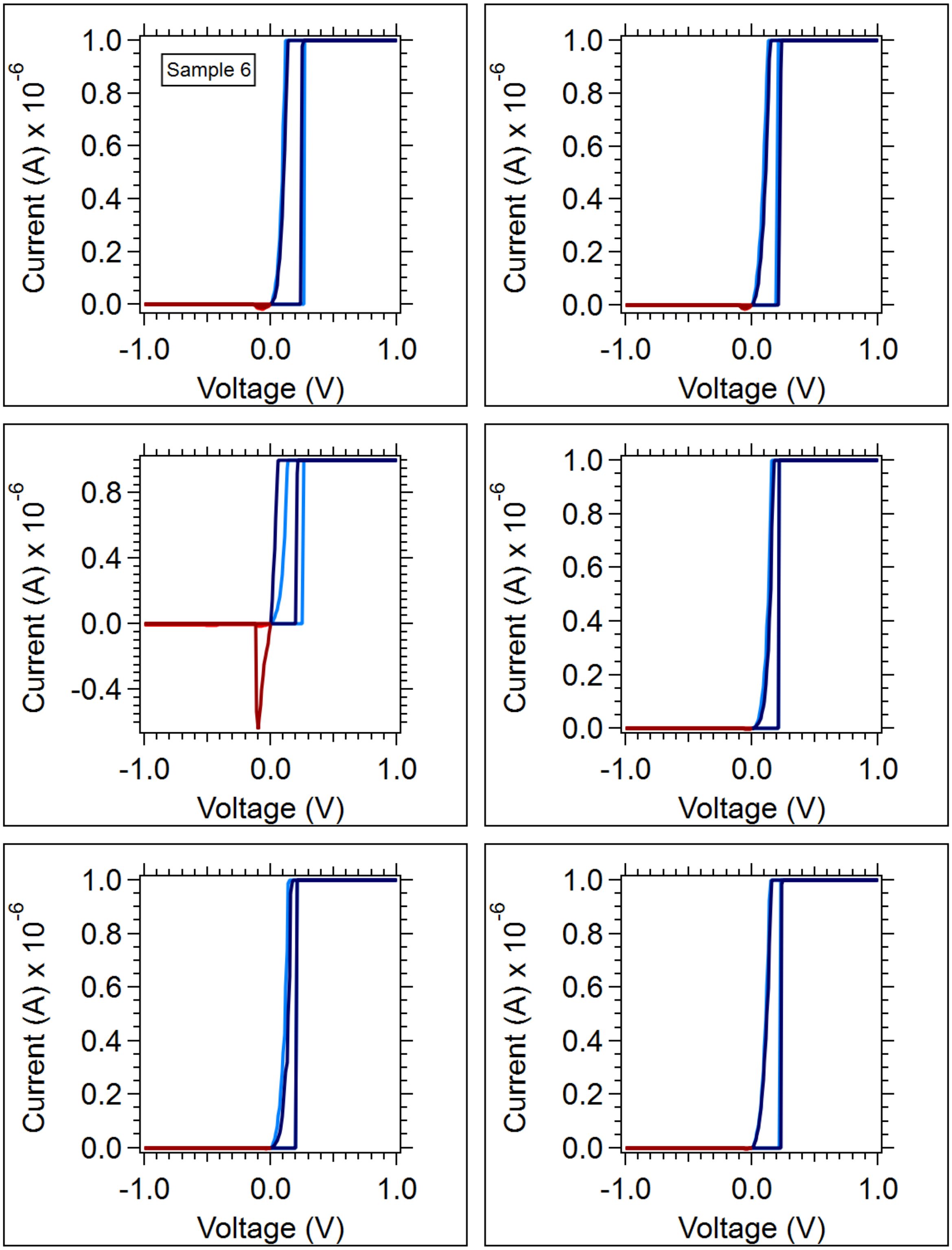

Figure S6a. DC data for measured Sample 6 devices

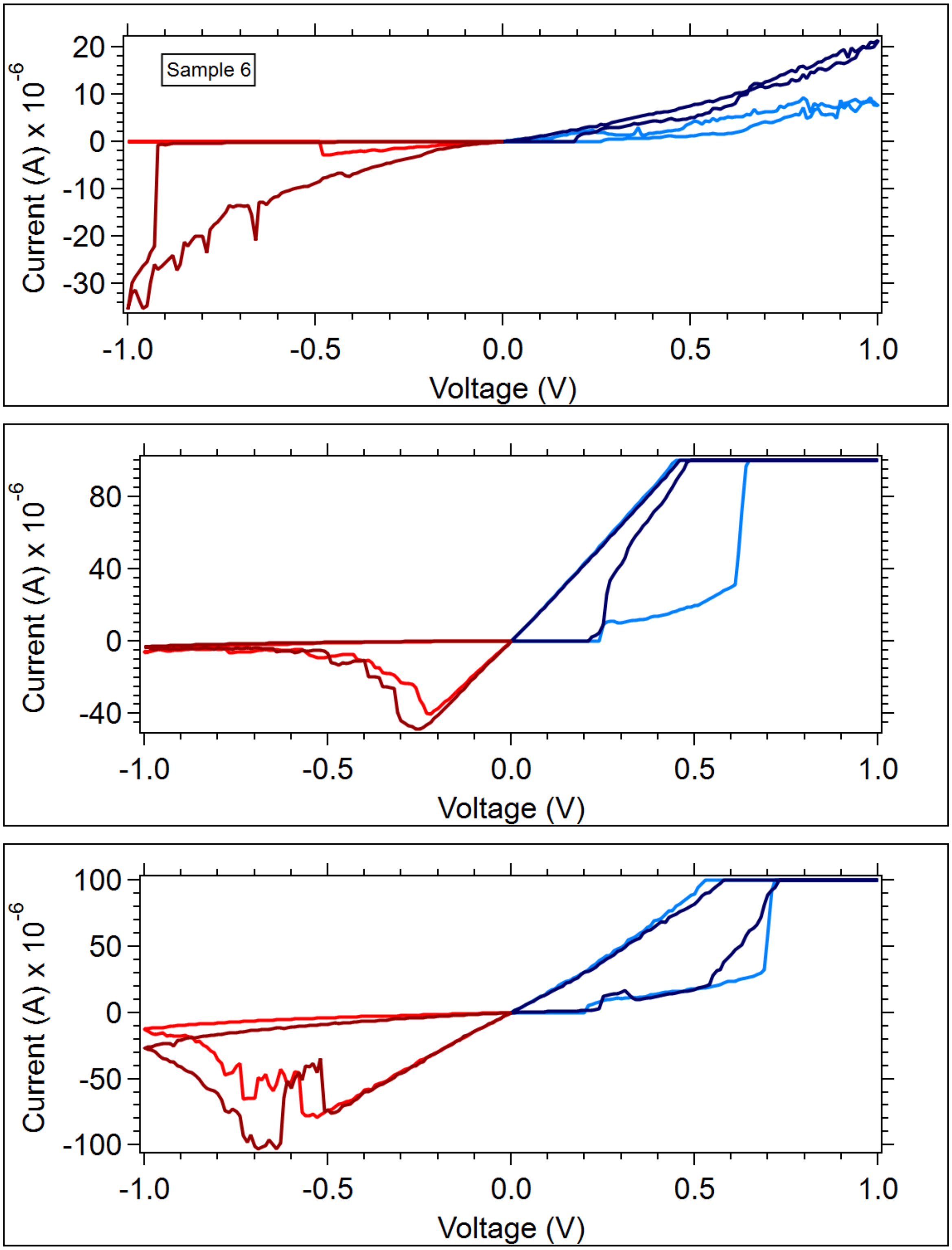

Figure S6b. DC data for measured Sample 6 devices

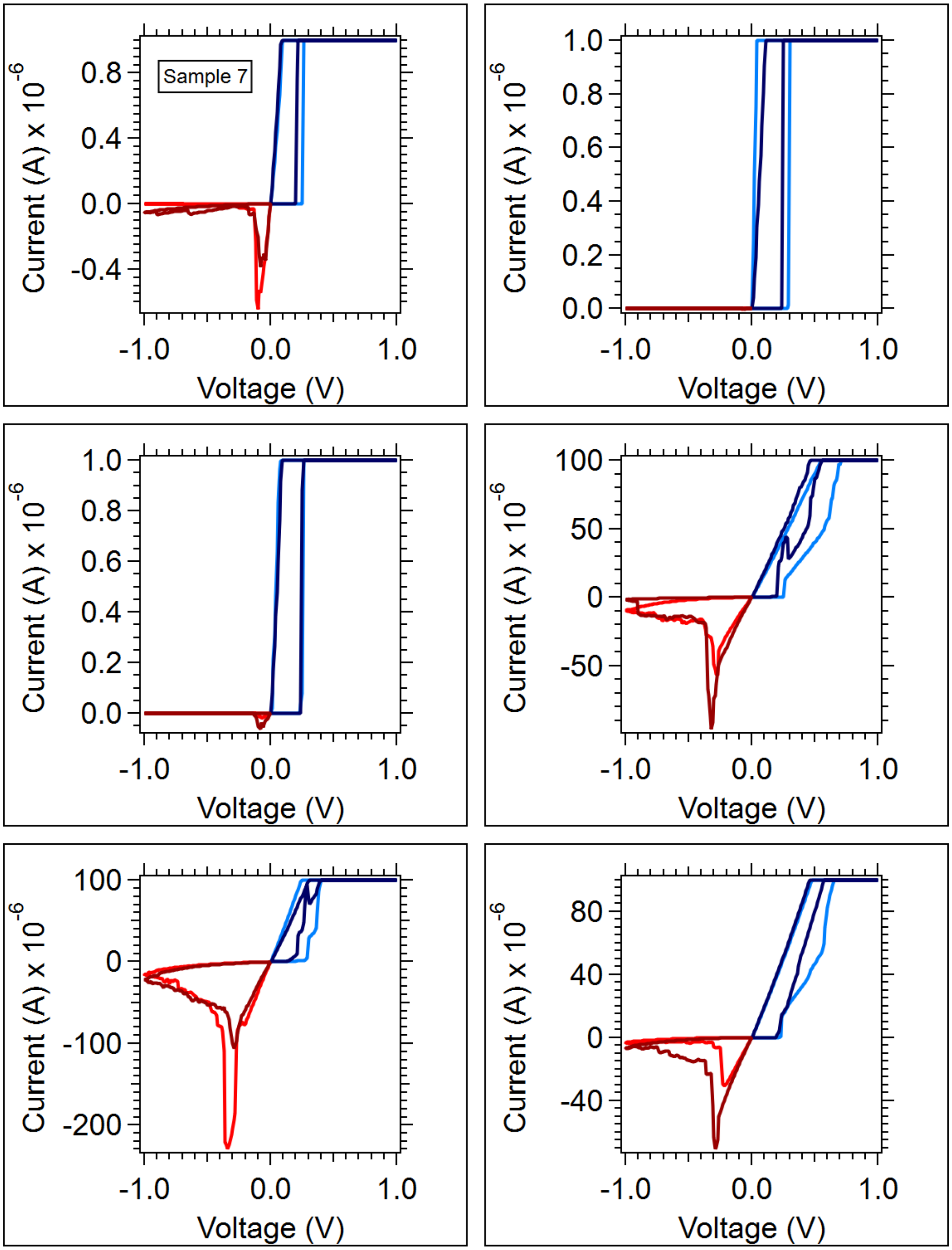

Figure S7. DC data for all measured Sample 7 devices

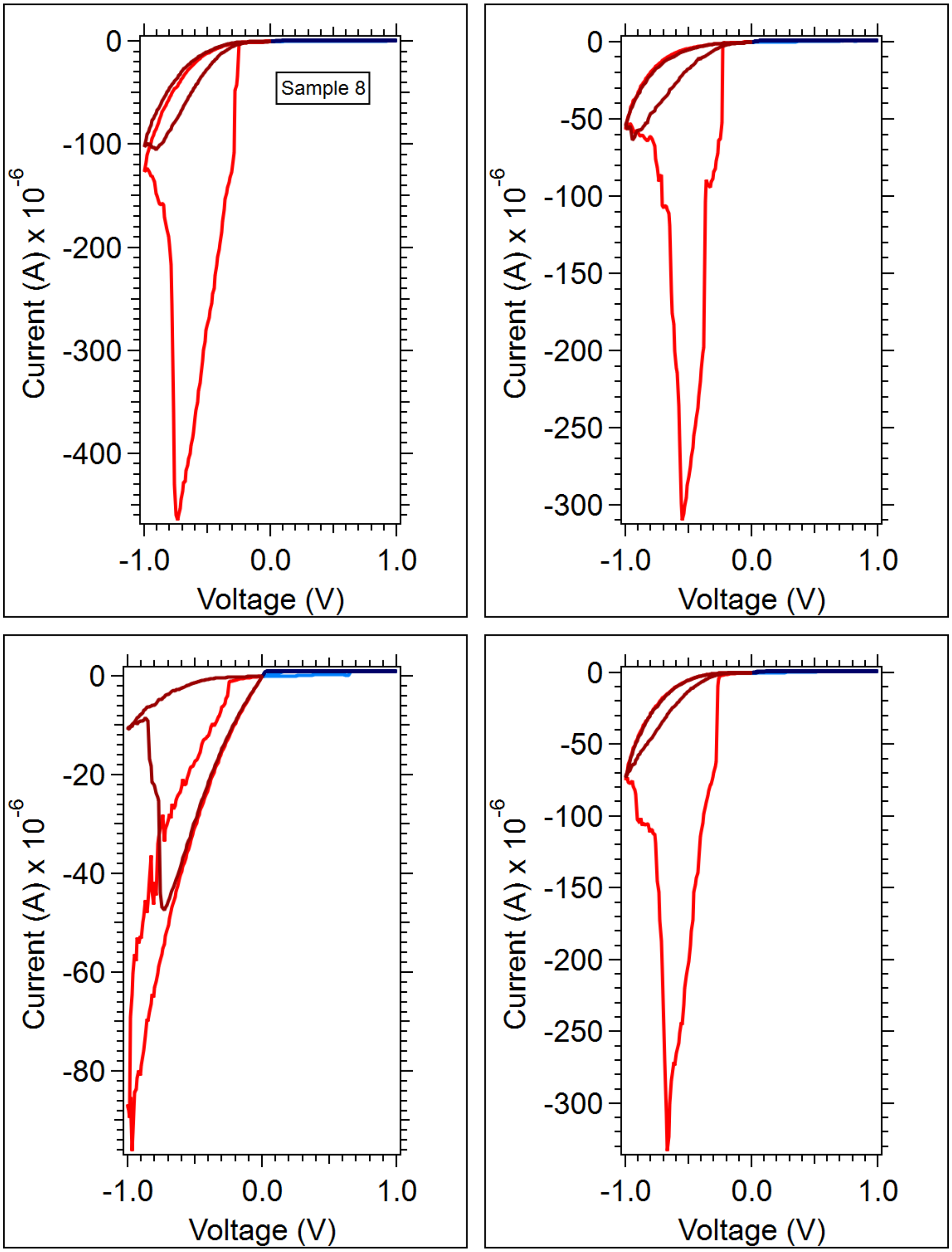

Figure S8. DC data for all measured Sample 8 devices

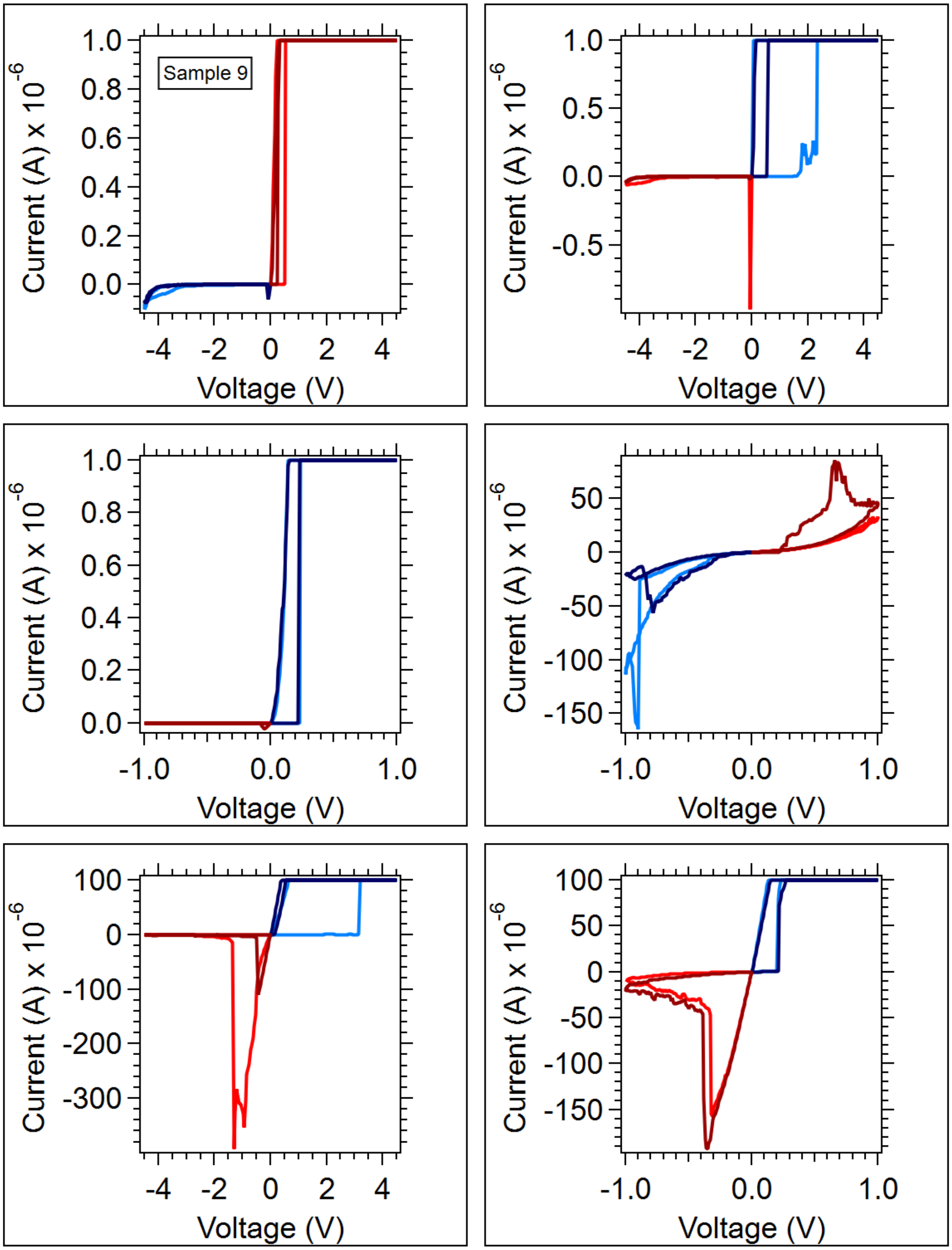

Figure S9a. DC data for measured Sample 9 devices

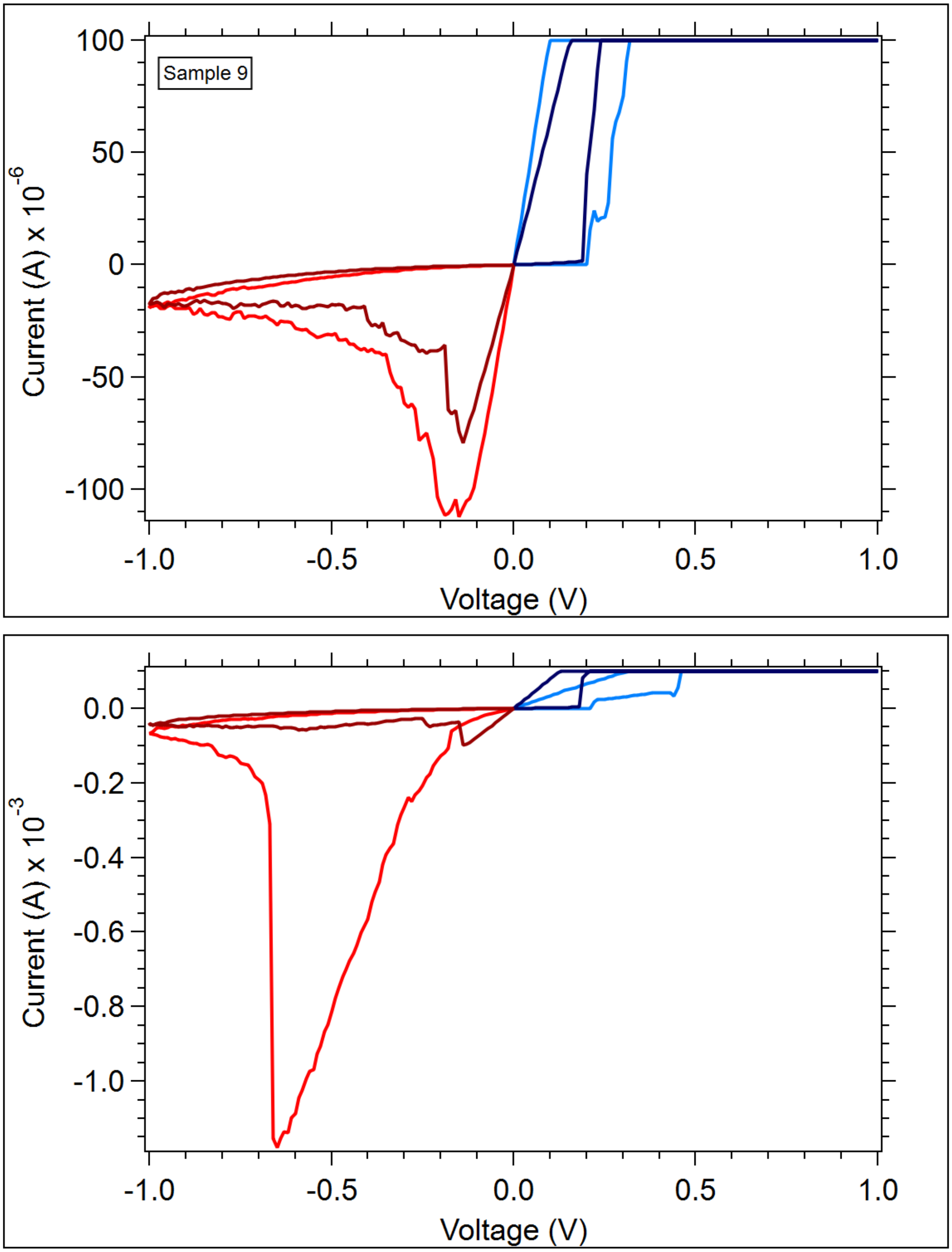

Figure S9b. DC data for measured Sample 9 devices

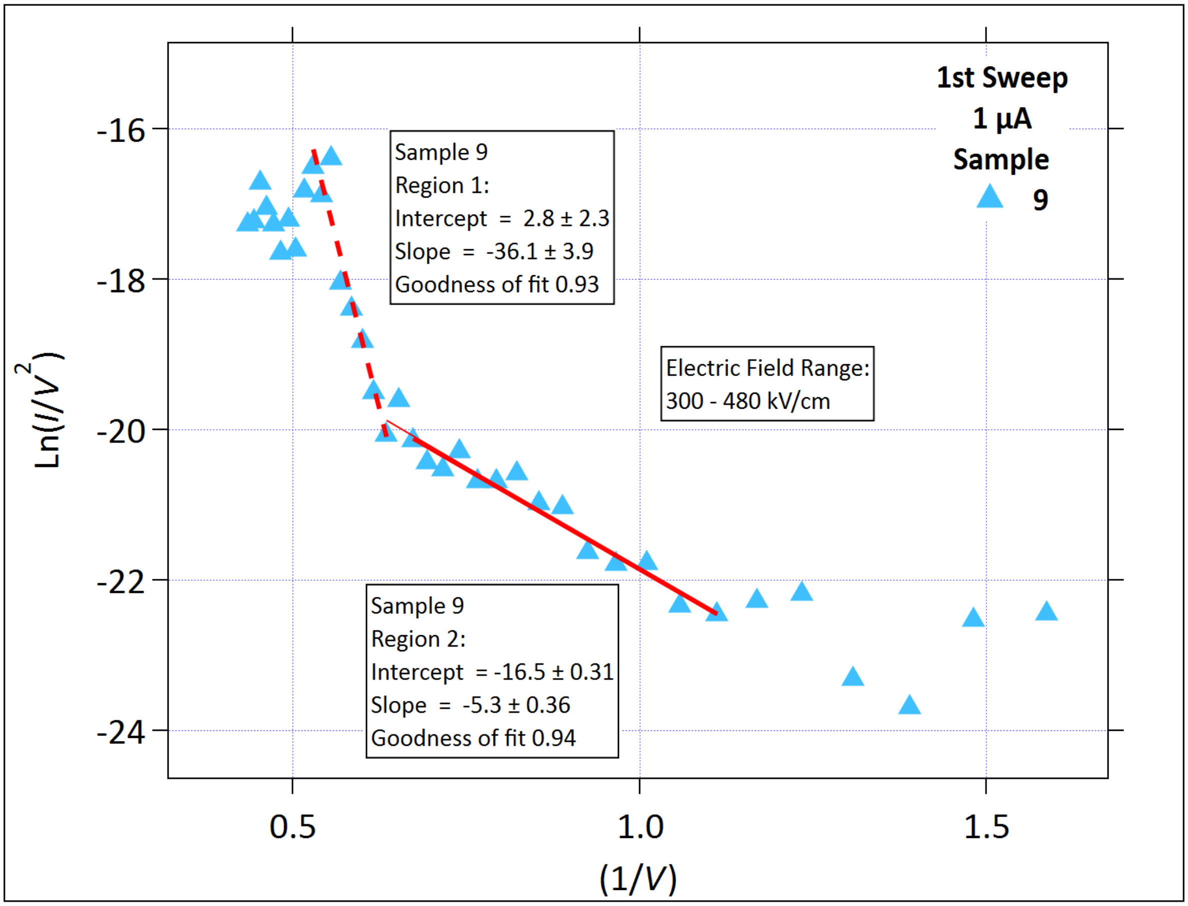

Figure S10. F-N plots for Sample 9
